# Post-diagnostic support in Australia: Perspectives of people recently diagnosed with dementia and their carers

**DOI:** 10.1177/14713012251333880

**Published:** 2025-04-14

**Authors:** Slađana Pavković, Lynette R Goldberg, Maree Farrow, Jane Alty, Melissa Abela, Lee-Fay Low

**Affiliations:** Wicking Dementia Center, 60119 University of Tasmania Faculty of Health, Hobart, TAS, Australia; Wicking Dementia Research and Education Centre, 60119 University of Tasmania College of Health and Medicine, Hobart, TAS, Australia; Wicking Dementia Research and Education Centre, 3925 University of Tasmania, Hobart, TAS, Australia; 4538 University of Sydney Faculty of Health Sciences, Lidcombe, NSW, Australia

**Keywords:** dementia, post-diagnostic support, people with dementia, carers, memory, family

## Abstract

*Introduction:* Timely post-diagnostic support is necessary to help people with dementia and those who provide care adapt to the diagnosis and optimise independence and quality of life. However, evidence from people with dementia and carers regarding the support they need is scarce. *Aim:* To explore the perspectives of people with dementia and their carers regarding the support they had been offered, barriers and facilitators to accessing such support, and support they viewed as desirable or ideal. *Method:* This was a cross-sectional qualitative exploratory study. Data were collected from individual interviews with 13 people recently diagnosed with dementia at nine Australian memory clinics and 17 carers. Interviews were conducted between September 2021 and October 2022. Reflexive thematic analysis was used to examine participants’ comments under four topics: Current Care and Support, Barriers, Facilitators, and Desirable Care and Support. *Results:* Current Care and Support: Four themes emerged: Communication with empathy; Written information valuable but could be overwhelming; Referrals provided but not necessarily followed; A focus on prescribed anti-dementia medications. Under the topic of Barriers, three themes were derived: Dementia stigma restricts life after diagnosis; Disapointment in Health and Aged Care systems; Limited insight into and/or difficulty expressing own needs. Under the topic of Facilitators, comments focused on Support from family and friends is crucial. Under the topic of Desirable Care and Support, three themes emerged: A single person guiding ongoing post-diagnostic support; Support with driving concerns; Engaging and tailored information. *Conclusion:* Perspectives of people recently diagnosed with dementia and their carers identified the need to ensure that post-diagnostic support addressed their individual needs, was clear, ongoing and involved the support of family and friends to reduce barriers and facilitate access. The importance of guidance by a trusted professional support person was considered key in facilitating more effective post-diagnostic dementia care.

## Introduction

Approximately 421,000 people in Australia live with dementia, with around 6.1 million providing support as informal carers or health professionals (Dementia Australia, 2023). Recent reviews highlight the need for immediate post-diagnostic support (Curnow et al., 2019; Miles et al., 2020; Morrisby et al., 2018; Soong et al., 2020). However, current support often focuses on medications rather than multidisciplinary services and non-pharmacological interventions, and varies between urban, regional, and remote settings (Pavković et al., 2024). Further, community-based aged and disability support services for dementia are frequently reported as difficult to identify and navigate (Xie et al., 2023).

People living with dementia expect more tailored support from health organisations, especially from services where the diagnosis is obtained (Morrisby et al., 2018; Steiner et al., 2020). In Australia, people aged 65 and over are eligible for government support via My Aged Care, which provides home support and residential aged care services (Moore, 2021). These services include assistance with household tasks, personal care, and access to specialist support such as nursing. Social support services facilitate community engagement, including help with shopping, recreational outings, and transportation. For those under 65 with disability and dementia, the National Disability Insurance Scheme (NDIS) provides home support services, but concerns exist about the lack of person-centred dementia support (Cations et al., 2022). Both older and younger people with dementia face difficulties accessing support, including service approval, assessments, choosing providers, and receiving appropriate and tailored support (Cations et al., 2022; Van Weel et al., 2019).

The main organisation in Australia that specialises in dementia support is Dementia Australia. This is the national peak body for Alzheimer’s disease and other dementias, offering a variety of services for people living with dementia, carers, and health professionals (Dementia Australia, 2022). These services include participation in psycho-interventional group programs like *Living with Dementia*, which offer group counselling and education for people living with dementia and their carers, along with follow-up counselling available in person or by phone. Specialised courses providing information about dementia trajectory and all associated changes in family relationships are also available exclusively for carers (Dementia Australia, 2022a). In 2020, Dementia Australia introduced a new post-diagnostic program comprising six sessions with a single professional point of contact (Dementia Australia, 2022c). These sessions are flexible and can be utilised at any time during the first year following diagnosis. The program aims to enhance understanding of dementia, identify support services, and prepare people living with dementia and their carers for the personal and life changes that can accompany dementia (Dementia Australia, 2022c). However, there is uncertainty regarding the extent to which people diagnosed with dementia receive information about the programs and how effectively they are implemented into practice (Pavković et al., 2024).

The most robust research evidence on the benefits of post-diagnostic support for people with dementia relates to the support provided by memory clinics both within Australia and internationally (Abley et al., 2013; Banerjee et al., 2007; Giebel et al., 2020; Meeuwsen et al., 2014; Mehrani & Sachdev, 2022; Naismith et al., 2022; Pavković et al., 2024). Memory clinics offer multidisciplinary services dedicated to dementia assessment, diagnosis and post-diagnostic care and support (Naismith et al., 2022). Of particular significance is evidence from the UK, which illuminates the breadth of services provided either directly by memory clinics or through facilitated connections to other support services (Abley et al., 2013; Banerjee et al., 2007; Field et al., 2021; Giebel et al., 2020; Killen et al., 2022; Morgan, 2021). The level of support can vary depending upon geographical area, but memory clinics in the UK have often provided psycho-education courses for people living with dementia, such as Living Well with Dementia (LivDem) (Woodstoke et al., 2024), psycho-social interventions (e.g., counselling, peer support), and cognitive stimulation therapy, tailoring information to individual circumstances and providing a link to an admiral nurse, link worker or dementia adviser (Abley et al., 2013; Banerjee et al., 2007; Giebel et al., 2020).

An early UK study evaluated the post-diagnostic support provided by the *Croydon Memory Service*, including prescribed medications, written diagnostic feedback, and tailored information. The evaluation showed reduced behavioural disturbances and improved quality of life for people with dementia after six months (Banerjee et al., 2007). Abley et al. (2013) examined the impact of peer support, cognitive strategy training, and carer education in dementia clinics in England. All were valued but unclear information hindered adaptation to life with dementia. Giebel et al. (2020) evaluated the services of a North West England memory clinic, which had adapted its post-diagnostic services to meet the needs of people with younger onset dementia (YOD). Findings showed that recipients valued counselling but found the psycho-social and cognitive interventions were insufficiently tailored.

Information about memory clinic services in Australia and their impact is based on the perspectives of health professionals (Mehrani & Sachdev, 2022; Naismith et al., 2022; Pavković et al., 2024). While valuable, it is imperative to include the perspectives of people with dementia and their carers to comprehensively capture objective data regarding the post-diagnostic support provided, as well as its timeliness, acceptance and accessibility. Including the voices of those directly impacted by dementia ensures that support programs and interventions are grounded in the reality of lived experiences, fostering more responsive and inclusive approaches to dementia care within the Australian context.

The focus of this study was *to explore the views of people recently diagnosed with dementia in memory clinics and their carers about post-diagnostic support*^1^ *provided to them, and their needs following dementia diagnosis.*

The questions for this study were:

1) What support was offered to people with dementia and their carers in Australian memory clinics in the year following the diagnosis?
2) What do people with dementia and carers think are the barriers and facilitators to accessing and utilising post-diagnostic services?
3) What do people with dementia and carers think should be ideal post-diagnostic support offered by memory clinics in the first year following diagnosis?

## Methodology

This cross-sectional qualitative exploratory study utilised semi-structured interviews, chosen for their flexibility in question delivery and response, to effectively explore the perspectives of people with dementia and their families (Manthorpe & Samsi, 2020). This approach is an established research practice employed by other researchers when engaging with participants with dementia (Field et al., 2021; Innes et al., 2014). The interviews conducted with carers adhered to a high level of sensitivity, particularly concerning their emotional well-being, while addressing explicit questions regarding the support they had been offered, used, and desired.

The paper focuses on data from 30 individual interviews (13 people living with dementia and 17 carers) conducted between September 2021 and October 2022. The study was approved by the Human Research Ethics Committee of the University of Tasmania (ref number-HC23946) and the Human Research Ethics Committee of Alfred Health Victoria – Multisite Acceptance Scheme (ref number- HC77604).

### Recruitment

We invited staff at 14 multidisciplinary memory clinics registered with the Australian Dementia Network (ADNeT) to assist with recruitment. ADNeT is funded by the Australian National Health and Medical Research Council to improve diagnosis, facilitate access to memory clinics, and enhance post-diagnostic support (Australian Dementia Network, 2021). Of the 14 clinics invited, 12 accepted, but only nine proceeded with recruitment. Three clinics were unable to assist due to the COVID-19 pandemic. Invitations to potential participants diagnosed with dementia within the previous year were sent by clinic staff. The Participant Information Sheet and Consent Form were emailed or posted by the first author (SP) to individuals who expressed interest. Consent was obtained via the Qualtrics online platform or by email/post.

Participants with dementia were informed that their support partner could be present at the interview to avoid separation anxiety. However, they were told that the research team preferred to engage separately with participants and carers to ensure each voice was clearly heard.

The semi-structured questions are listed in Appendix 1.

### Data collection and analysis

All consenting participants were called before their scheduled interview to discuss the study, answer questions, and minimise any risk of distress. The semi-structured interviews allowed participants recently diagnosed with dementia to discuss their health and memory-related concerns. This approach ensured that participants were not compelled to discuss uncomfortable topics. Emphasis was placed on their recollection of the diagnosis, experiences living with dementia, and emotional responses. The interviewer (SP) used terms like “dementia” and “remember” only after participants did, respecting their comfort level.

All interviews were audio recorded and transcribed via Zoom, with manual corrections by the first author (SP). Reflexive thematic analysis was applied to the data using NVivo software. The six-stage approach (Braun & Clarke, 2006) allowed for flexible, exploratory analysis. Two authors independently coded the transcripts to enhance credibility. The interpretation of codes and development of draft themes were completed by the primary and senior authors (SP & L-fl). Themes and subthemes were found collaboratively by all authors, with regular discussions incorporated into the final analysis and themes, consistent with the principles of reflexive thematic analysis (Braun & Clarke, 2019).

## Findings

### Interviews

Two of the 30 interviews were conducted face-to-face, 17 via Zoom video conference and 11 via telephone. The restrictions relating to COVID-19 prevented face-to-face interviews for most participants. Phone calls were connected to Zoom to enable recording and transcription. The average time between diagnosis and interview was eight months. The interviews lasted between 15-80 minutes; the average length was 41 minutes.

### Participants

All participants were from four clinics in Victoria (VIC) (three public, one private), two clinics from South Australia (SA), one clinic from Queensland (QLD), one clinic from Tasmania (TAS), and one clinic from New South Wales (NSW) (see Table 1). Eight clinics were public, and one was private; five were in metropolitan and four in regional areas. Of the 30 participants, 23 were 65 years of age or older (10 people with dementia, 13 carers); 3 were people with YOD (less than 65 years of age) and 4 were carers looking after people with YOD. Of the participants with dementia, 3 were from culturally and linguistically diverse (CALD) backgrounds. Table 1 summarises demographic data and the type of each clinic.

**Table 1.** Participant demographic and clinic data.

Location	Number and type of memory clinic (regional/metropolitan, private/public)	People with dementia	Carers	Gender
Victoria (VIC)	3 regional/public, 1 metropolitan/private	3	7 (2 for people with YOD)*	3 males, 7 females
Queensland (QLD)	1 metropolitan/public	2	2	1 male, 3 females
South Australia (SA)	2 metropolitan/public	4 (1 with YOD)*	5 (1 YOD)*	3 males, 6 females
New South Wales (NSW)	1 metropolitan/public	2 (1 with YOD)*	3 (1 YOD)*	2 males, 3 females
Tasmania (TAS)	1 regional/public and research	2 (1 with YOD)*	No carers	1male, 1 female
Total	5 metropolitan, 4 regional; 1 private, 8 public	13 (3 with YOD)*	17 (4 caring for people with YOD)*	10 males, 20 females

*YOD = Younger Onset Dementia.

### Identified themes

Four themes were derived under the topic ***Current Care and Support***: *Communication with empathy; Written information valuable but could be overwhelming; Referrals provided but not necessarily followed; A focus on prescribed anti-dementia medications.*

Under the topic of ***Barriers,*** the following themes were derived: *Dementia stigma restricts life after diagnosis*; *Disapointment in Health and Aged Care systems; Limited insight into and/or difficulty expressing own needs.*

**Facilitators** *were identified as: Support from family and friends is crucial.*

**Figure 1.**
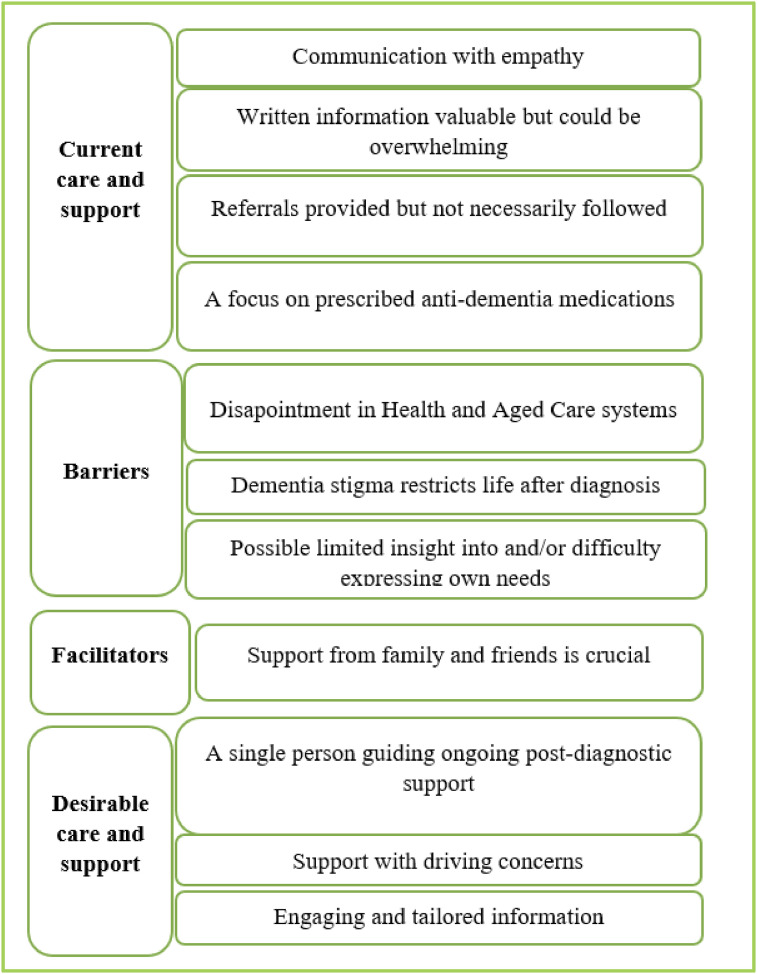
Four identified topics and themes derived from participants’ responses to three research questions.

Three themes were derived under ***Desirable Care and Support***: *A single person guiding ongoing post-diagnostic support; Support with driving concerns; Engaging and tailored information* Figure 1.

#### Current Care and Support

##### Communication with empathy

Apart from participants who attended one clinic that operated as a “one-stop” diagnostic clinic, all participants saw their specialist and memory clinic staff at least twice. Regardless of how many appointments they had, participants reported that the communication discussing the diagnosis and other information was appropriate, and delivered with empathy, which was invaluable. The following comments are reported as spoken:

“The doctor has been very, very empathetic and kind, so it hasn't been trauma…That was what we needed in disclosing such bad news**” (carer, female, SA)**

Many participants referred to the news about diagnosis as bad news and shock, but the approach of staff and specialists in memory clinics was highly valued.

“It was a shock, but doctor was empathetic and give us time to proceed, also show the pictures from the brain and it helps a lot**” (carer, male NSW)**

##### Written information valuable but could be overwhelming

Although carers perceived the entire process of diagnosis, communication of results, and delivery of information about post-diagnostic support as being conducted with empathy, they struggled to recall the specific purposes of the services. While they appreciated the empathy shown, they also expressed confusion regarding other matters discussed or provided.

“The doctor was amazing, and the nurse had a gently approach, but they talked about unfamiliar things, and gave us a folder with papers. However, it was too much for us and we were left confused about our next step” **(carer, female, VIC)**

Information about dementia and services was provided to all participants. While a few participants with dementia described receiving “nothing,” their carers reported receiving ample, frequently overwhelming, written information.

Resources from Dementia Australia aim to be clearly written with the focus on strategies in communicating about dementia, and how to adjust to potential changes in relationships with family and friends. One of these resources was cited by a person with dementia as something that she could still remember as valuable. She stated:

“I read a booklet “Family and Friends”, I remember that” (**person with dementia, female, QLD)**

However, many participants talked about not being able to find a way through the provided information and that they were confused.

“They generously provided a package of information. |However too many papers with plenty of words that no one explained discouraged me to open the package. I may try to go through once, but now I have no time for this” **(carer, female, VIC)**.

##### Referrals provided but not necessarily followed

While some participants were unsure of the precise name of the organisations to which the memory clinic referred them, they recalled that Dementia Australia (DA) was mentioned and received written information about this organisation. Several participants reported positive experiences with memory cafés—social gatherings where people with dementia and their carers could meet in a public setting, exchange information, and learn about available services from each other and a café facilitator. Similarly, carer support groups, led by a trained facilitator with expertise in dementia and counselling, provided carers with the opportunity to share experiences, receive emotional support, and access practical advice.

“Memory clinics eventually push you to somewhere else and to Dementia Australia. Memory café suits me, I met there some people, very nice people” **(person with dementia**, **VIC)**

However, the majority of participants had not engaged with these services, often due to a lack of awareness, accessibility issues, or personal preferences. Some carers expressed difficulties in finding suitable support groups, while others felt that their caregiving responsibilities or time constraints prevented participation. These findings highlight the need for greater outreach, flexible service delivery, and tailored support options to encourage broader engagement with these beneficial resources.

One reason that participants with dementia may have refrained from reaching out to organisations like Dementia Australia may have been their perception that they did not need such social support.

“We got a package with information, and I remember some leaflets from Dementia Australia, but I have not ever been interested in social support” **(carer, female, QLD)**

At the memory clinic in Tasmania, a representative from Dementia Australia was present at the post-diagnostic session when the diagnosis was discussed. However, neither of the two participants from this memory clinic had accessed the post-diagnostic support programs offered . They explained that it was too early for them, they were planning to contact Dementia Australia but had other priorities, or they were not certain about the purpose of these services.

“I met a person who was there because of support, I think she mentioned Dementia Australia, but I did not contact her, not yet. I think I am in very early stage and I do not need that support, but I would like to have at least phone follow-ups. If someone at times check on me it would be enough for the beginning.” **(person with dementia, female, TAS)**

##### A focus on prescribed anti-dementia medications

Most participants were prescribed anti-dementia medications soon after diagnosis. Some considered this as the only professional care provided to them following their dementia diagnosis.

“Yes I am taking Aricept, it was firstly mentioned by a doctor who diagnosed me, but I was discharged, and then my GP continued. I am fine” (**person with dementia, male, VIC**)

Since most follow-ups for medications were conducted by General Practitioners (GPs), this encouraged participants to see their GP. Participants expected their GP to be engaged holistically in their care and support. However, many expressed concerns that their GP did not provide comprehensive post-diagnostic support:

“My GP prescribed only medications and he said not to worry as I am in the early stage of dementia, but it was not very helpful” (**person with dementia, female, SA**)

While prescribed medications were typically perceived as an intervention to be followed under a doctor’s supervision, the needs of many people diagnosed with dementia extended beyond pharmacological interventions.

“Our GP did not understand that I need more than medications for my wife. I know that medication will not give her a cure, so I wanted to know about some aids. I know that there is a GPS for tracking a person … but he made me feel uncomfortable asking any questions” **(carer, male, NSW).**

#### Barriers

##### Dementia stigma restricts life after diagnosis

One frequently identified barrier to access post-diagnostic services was the stigma and negative emotions that continue to be associated with the condition. Despite being aware that dementia is caused by brain changes, some participants described feelings of shame and insecurity. They expressed discomfort in unfamiliar environments and worried about encounters with people who they may not recogn ise or recall. The heightened frustration experienced in public exacerbated their sense of self-doubt and fear of rejection. Several comments underscored these feelings, highlighting the persistent stigma associated with dementia.

“I cannot …accept myself, I do not want to meet new people and cannot introduce myself and meet familiar people and cannot recognise them” (**person with Younger Onset Dementia, female, NSW)**

“I do not want to go to the (memory) café with strangers. For me, seeing my friends is not easy too…I just do not want to leave my house…I am not happy, but I am safe, I cannot be underestimated if I am home” **(person with dementia, female, VIC)**

“I do not want to share my story with people I do not know, even they have similar stories” **(person with dementia female, SA)**

“I have not shared my diagnosis with my friends. They are all forgetful and no one says that they have dementia. If I say I have dementia they will turn back on me” **(person with dementia, male, VIC)**

The fear of rejection, even by friends, and the frustration of feeling a loss of dignity were described by many participants with dementia.

##### Disappointment in the health and aged care systems

Participants expressed concern about the inefficiency of the health system, particularly in the lack of continuation of support and the need to pay out of pocket for most post-diagnostic services. One of the carers commented that a driving test cost them $700. Concerns also related to services offered through *My Aged Care* as reflected in the following:

“You go on the website [My Aged Care] and you look for service provided that along with cleaning, transport, meals on wheels, offers a specialist dementia service, as we were told about this option at our assessment, but you do not get a dementia specialist you got a manager who really do not know enough about dementia” **(carer, male, NSW)**

Similar frustration was reported with the NDIS:

“We did not get NDIS, we did not have anyone who could help us to get it. He was too good for them, but he is not. He tried his best to impress them because he is ashamed that he got dementia” **(carer, YOD, female, SA)**

Carers who were knowledgeable about cognitive interventions expressed dissatisfaction with the lack of subsidies for such services in the Australian system. They believed that the healthcare system needed to offer ongoing support with cognitive interventions considered within the range of allied health services, thereby warranting financial assistance. A participant with dementia commented:

“We need a more human health system. I was working for this Government my whole life, and now when I cannot work, I need to pay everything, each home service I got…there is not a free service” **(person with dementia, male, VIC)**

Participants acknowledged that the key role of memory clinics is primarily diagnosis rather than post-diagnostic support. However, they expressed a strong belief in the necessity of being connected to additional services following diagnosis, and they placed responsibility on the government for not structuring the healthcare system to alleviate and maintain such connections.

“I do not think that people in memory clinics need to explain to us where to go, everyone there was busy. However, if not there, you need to know where you can find the support. Health system is not organised well” **(carer, female, VIC)**

##### Limited insight into and/or difficulty expressing own needs

People with dementia and some carers appeared to experience difficulty identifying or expressing their needs or wishes. Many with dementia spoke generally about experiencing forgetfulness and being on medication to alleviate symptoms but did not specify particular needs. During interviews, they often redirected conversations away from their diagnosis, consistently responding with ‘I’m okay, I’m happy.' When directly asked about their dementia, some acknowledged it as early Alzheimer’s, while others listed various unrelated illnesses and surgeries from their past. Carers were aware of the information available to them but indicated that they currently did not require any additional help or services, as the support they received from their children and extended family, or friends was deemed sufficient.

#### Facilitators

##### Support from family and friends is crucial

Participants, particularly carers, emphasised family support as most important in the process of receiving a diagnosis of dementia and learning about accessing post-diagnostic support. Only one carer stated that she and her husband did not want to share the diagnosis with their children. The carer said:

“I did not want for them to worry and feel that they need to take something from their young family and give to us” **(carer, female, NSW)**

Others mentioned that sharing the diagnosis with their family helped them feel secure. Family members’ understanding, efforts in seeking information and adequate services, and spending time with the person with dementia to provide relief were identified as significant sources of support after discharge from memory clinics. Some comments follow:

“I am so lucky my family has been amazing since my husband got the diagnosis” **(carer, female, QLD)**

“If I did not have a family, I would feel so alone. My daughter is a psychologist, and she helps a lot” **(carer, female, SA)**”

“Our daughter found a carer support group for me. I looked at the website Dementia Australia, but I am not very good in that stuff. I thought I should call DA and I called and got a package. Oh, it annoyed me…then my daughter called some numbers and organised for me a carer support group. It is very good, but I did not know that I needed it **(carer, female, NSW)**.

Immediate and extended family members also facilitated post-diagnostic support and care and provided information about available services, links to such services, and navigation of such services. Family support was particularly evident in organising social, physical, and leisure activities and ensuring such activities became routine. In addition, one of the participants (carer) referred to the cognitive intervention attended by his wife that was organised by their son:

“Our son knew Dr X who is a neuropsychologist providing computing cognitive interventions. My wife attended 12 sessions and it was a turning point for us. Dr X assessed her after first 12 sessions and decided to do another 12 sessions. We were so lucky as our son knew Dr X. We never heard about that intervention from any of the doctors that were included in the previous support of my wife” **(carer, male, VIC)**

This intervention consisted of 12 sessions with a neuropsychologist, focused on computerised cognitive training. The neuropsychologist assessed cognitive function before and after the initial 12 sessions. If improvement in cognitive function was observed, as it was in this case, an additional 12 sessions were recommended. The goal was to enhance cognitive abilities through targeted exercises, adapting the intervention based on individual progress.

#### Desirable care and support

##### A single person guiding ongoing post-diagnostic support

Carers strongly advocated for a dedicated, knowledgeable person who would ensure continuity of services, explain the best options available from the outset, and work with people with dementia and carers to adapt these options as the needs of each individual evolved with the progression of dementia. Participants, both carers and people with dementia, expressed a desire for someone who could assist them in accessing tailored person-centred support, guiding them seamlessly from the point of diagnosis through the various stages of dementia and helping them obtain appropriate care and support. Many described this person as a central professional point of support, someone “we can always call”. This what one of the carers said:

“I need somebody who could help me to understand the purpose of the institution and organisation for support and it is good if I can discuss with that person different stuff relating to my husband’s care to tell me who to call and what to say if I am not certain about something” **(carer, female, VIC)**

##### Support with driving concerns

Being able to continue to drive was a recurring theme raised by many people living with dementia, while carers also expressed concerns about driving-related issues, such as persuading their loved ones to stop driving, hiding the keys, or dealing with their unhappiness about not being able to drive. Being able or unable to drive was identified as a significant challenge by both people living with dementia and carers and highlighted the value of working with a single person as a professional point of contact.

The following quote from a person with dementia reflects similar comments from others regarding the need for a guide:

*“*We got information for driving test to book. However, I did not know what to say when I call them, how much it will be, what to expect there. They all say ‘driving test’ and I know if I lose my driving that’s the end. I do not want to lose it, I want to do my best, but I need a person who can help me, explain to me, guide me… And all other information, I want someone who can be my guide, like I had a nurse when I had a cancer” (**a person with dementia, female, VIC)**

Discussions about driving elicited anxiety due to the complexity of the situation and the perceived difficulty in finding a resolution.

“I may not know what I want, but I know what I do not want - I do not want giving up driving” **(person with dementia, male, SA)**

Driving was seen as a sign of independence and losing this was equal to losing their sense of self. A person living with YOD commented:

“But I can't do… like if you go to the shop, I have to take the girls to drive me and be with me. Yes, if I wanted to buy something that I needed for me, for myself, underwear, I need to have my girls or husband with me” **(person with dementia, female, NSW)**

An older person with dementia confirmed that losing a driving licence means a loss of independence:

“I know I have a problem with memory, but I had no problem with driving, I never had. However, they took away my driving licence and destroyed me. They just told me to stop to drive. If you return driving licence to me you will return my life back” **(person with dementia, male, VIC)**

The complexity of this driving issue transitioned from a barrier to maintaining independence to the need for desirable support to help with the driving assessment. This included having someone who could explain driving test requirements, as well as provide counselling both before the test and afterward if the driving license was revoked or restricted to a 15 km radius within the local area. Some participants had been told to stop driving, without any test or preparation for this impactful change in their lives.

##### Engaging and tailored information

As noted under Current Care and Support, many carers expressed frustration with the information they had received, commenting that the abundance of information made it challenging to discern the relevance of each document to their needs. This highlighted the importance of information to be clear, concise, engaging, and presented in multiple ways to suit the recipient, for example presented in stages with easily recogn isable pictographs.

“We got a folder full of papers, but I do not think this is all relevant for us. We need a few information for the beginning with a clear explanation of the purpose of each recommendation” (carer, female, VIC).

Many carers expressed a similar need for concise and engaging information:

“I wish to get a paper or a brochure that I can easily remember or when I want to check I can easily find the information” **(carer, female, VIC)**

## Discussion

People with dementia and carers identified that memory clinics had a focus on pharmacological therapy rather than providing information about a range of post-diagnostic services. This perceived emphasis on medications aligns with the perspectives of healthcare professionals (Banerjee et al., 2007; Fereshtehnejad et al., 2014, 2015; Meeuwsen et al., 2009; Mets et al., 2013; Pavković et al., 2024 ; Tsolaki et al., 2021). Carers mentioned receiving referrals to services provided by Dementia Australia and other organisations but often did not follow up, possibly due to confusion, lack of understanding, or feeling that the generic information did not address their individual needs. These reported barriers to accessing services align with international literature (Lim et al., 2012; Robertson et al., 2022; Zwingmann et al., 2020).

Additional barriers to accessing and utilising post-diagnostic support included the stigma associated with dementia, the need for time to adjust to the diagnosis, and difficulty expressing needs. Despite efforts by organisations like Dementia Australia to promote dementia-friendly communities and increase awareness of the condition (Alzheimer’s Australia, 2014; Phillipson et al., 2022), many participants chose not to disclose their diagnosis, appearing to confirm the persisting stigma. Stigma has been identified as a significant barrier to seeking help and accessing services post-diagnosis (Morrisby et al., 2018; Stites et al., 2018). These findings suggest the need for multiple sessions following a dementia diagnosis to address these issues. Programs like “Living Well with Dementia,” which consist of seven to 10 sessions, provide time to focus on discussions regarding acceptance of a diagnosis and access support services (Cheston et al., 2023; Woodstoke et al., 2024).

An additional reason for people’s reluctance to share a diagnosis of dementia and access services can reflect the time needed to work through the stages of grief after receiving such a diagnosis (Tyrrell, 2024). All participants in the current study had received their diagnosis in the previous 12 months. It is understandable that they needed time and assistance to work through the non-linear stages of grief such as shock and disbelief, searching and yearning, disorganisation and repair, rebuilding, and healing (Tyrrell, 2024). This time for grieving needs to be recogn ised and integrated in early conversations following a diagnosis of dementia.

All participants with dementia in this study received their diagnosis during the Covid pandemic. Although they did not explicitly mention the pandemic as a barrier to accessing services, it may have played a role. Some forms of social support, such as peer groups (e.g., memory cafés and carer support groups) and group education, likely were not available in certain areas due to pandemic-related restrictions. As a result, participants may not have been referred to these services. Research on the impact of Covid on people with dementia and their carers has highlighted consequences such as depression, anxiety, social isolation, loneliness, decreased quality of life, and increased burden of care, all of which can affect service access and engagement with post-diagnostic support programs (Giebel et al., 2021; Hicks et al., 2022; Messina et al., 2022). These findings suggest that restricted access to counselling, care support, and day centres during the pandemic may have significantly reduced respite opportunities for carers.

Uncertainty about and distrust of the health and aged care systems was a further barrier for participants in accessing post-diagnostic services. Carers acknowledged that current/limited post-diagnostic support did not meet their needs. They expressed dissatisfaction with the practical support (e.g. personal care, gardening, food preparation, medication monitoring, and specialist dementia support) provided through My Aged Care and its Home Support Program. Healthcare professionals have reported the same concerns (Moore, 2021; Phillipson et al., 2022). Carers of people with YOD face-to-face criticised the NDIS and its assessors for inadequately evaluating the level of disability and needs of individuals with YOD, resulting in minimal or no support. In 2022, Dementia Australia made a submission to the Joint Standing Committee of the NDIS with recommendations for changes to enhance understanding among agencies that provide support for people with YOD (Dementia Australia, 2022).

Post-diagnostic services such as cognitive intervention were reported as only available at private memory clinics, and thus only available for people who could afford to pay. Participants who accessed private memory clinics did so without the assistance or referral of a healthcare professional.

Both people with dementia and carers recogn ised family support as the most significant facilitator following a dementia diagnosis. It symbol ised trust, continuity, and assurance, enabling participants to adjust to the diagnosis and find enjoyment in non-pharmacological interventions like walking, gym activities, meeting friends, and going to the local club. The literature shows extensive evidence about the importance of families and family carers (Aldridge et al., 2019; Chrisp et al., 2012; Hua et al., 2021; Milne & Larkin, 2023; O'Shea et al., 2018; Zwingmann et al., 2020). The family presents a complex and flexible point of support that can fill many gaps. However, this raises several points for future discussion. It remains uncertain how individuals without accessible family support can manage life with dementia post-diagnosis. Additionally, financial compensation for the time family members spend in providing care needs consideration, along with respite care services to enable a break from care. Milne and Larkin (2023) discussed these issues, linking them to human rights concerns that have been overlooked until recently but now require urgent attention from policymakers and governments worldwide.

Focusing on ideal services, participants with dementia and carers identified the need to have ongoing contact with a trusted professional person to help them understand the healthcare system, identify and explain available services to optimise independence and quality of life, clarify the purpose and costs of such services, enable access, navigate them through their chosen services, and enable continuity of support to alleviate the burden on families and carers. These insights confirmed the findings of recent Australian studies (Pavković et al., 2024; Xie et al., 2023). Currently in Australia, terms and models for a single point of professional contact vary, including support manager, case manager, link worker, dementia advisor, dementia coordinator, and dementia nurse (Renehan et al., 2017). Regardless of which “case management/care coordinator” model is the best for dementia support, researchers, healthcare providers, and people with dementia and carers concur that a key point of contact is essential for appropriate, effective, and ongoing support (Goeman et al., 2016; Gridley & Parker, 2022; Harper, 2019; Iliffe et al., 2014; Pavković et al., 2024; Reilly et al., 2015; Van Mierlo et al., 2014; Vroomen et al., 2012; Vroomen et al., 2012, 2012).

Of particular concern to people with dementia, and carers, was the need to maintain their independence, to optimise their quality of life, freedom, and social interaction. A key aspect of this independence was a person’s ability to continue to drive (Chacko et al., 2015). This issue is well-documented in recent European and Australian studies (Chacko et al., 2015; Connors et al., 2018; Frederiksen & Waldemar, 2018; Gilliard et al., 2016; Liddle et al., 2016; Morrisby et al., 2018; Scott et al., 2023; Telenius et al., 2020). Concerns about driving emerged more prominently in the interviews with people with dementia compared to those with carers. Moreover, people with dementia and carers had different concerns regarding driving. People with dementia wanted to continue to drive while carers often wanted them to stop driving. Both cases highlight the need for clear and relevant advice in early and ongoing conversations about post-diagnostic support.

Participants stressed the need to receive information that was clear, engaging, not overwhelming, and tailored to individual needs, both in content and delivery. These findings align with sentiments from participants in previous studies (Merl et al., 2022; Pavković et al., 2024; Poyser & Tickle, 2019; Vince et al., 2017; Wollney et al., 2022).

### Strengths and limitations of this study

This globally relevant research affirms the importance of including people living with dementia in research to optimise their care. Strengths include a robust qualitative research methodology, commonly employed in similar contexts, that highlights the voices of people with dementia and carers in identifying desirable care and support post-diagnosis. Participants with dementia and their carers represented nine memory clinics from different states across Australia. While impactful, the findings of this study do not reflect the cultural perspectives of Aboriginal and Torres Strait Islander peoples and people from other culturally and linguistically diverse communities. A notable bias may also exist, as participants who engage in research tend to be more educated.

The semi-structured interviews were guided by questions designed to probe positive, challenging, and ideal experiences of participants regarding post-diagnostic support. The primary aim was to allow participants to share their stories and freely associate, uncovering their thoughts and intentions. Previous research showed that memory clinics in Australia provided focused but not comprehensive support and it was important to explore this further through the perspectives of people living with dementia. Some intended questions remained unasked as conversations naturally diverged in different directions. Some questions received responses from only one participant. When these single responses were aggregated with other data, they did not generate a distinct theme and were therefore not included.

This flexible approach ensured that the experiences of the participants shaped the findings, allowing for a rich and authentic exploration of their perspectives. While not all data contributed directly to the final analysis, the dynamic nature of the discussions provided valuable insights into the lived experiences of people with dementia and their carers.

## Conclusion

Insights from people recently diagnosed with dementia and their carers highlighted critical areas to address to strengthen the post-diagnostic support provided by memory clinics. A key priority is to ensure that the information provided, both verbal and written, is clear, engaging, not overwhelming, tailored to the needs of each person, and communicated with empathy. Further, people recently diagnosed with dementia in memory clinics and their carers should be offered the follow-up appointments. These appointments are needed to encourage people to attend for post-diagnostic support and explore the reasons why they may be apprehensive about doing so, including addressing the stigma that is often associated with dementia. Multiple sessions are needed to evaluate individual needs and wishes, that is the concerns expressed around driving and the need for independence. Post-diagnostic services need to be comprehensive, including both pharmacological and non-pharmacological interventions. Access to such services needs to be coordinated by a trusted key professional contact.

There is a pressing need for more public campaigns and dementia education to increase dementia literacy and reduce societal stigma. Such campaigns and education need to interface with government funding to compensate family members and friends who provide valuable support for which they are not yet paid.

Implementing such comprehensive, coordinated, and funded post-diagnostic services within the Australian public healthcare system will require structural changes at state, territory, and national levels. It is imperative to ensure that the perspectives of people with dementia, carers, healthcare professionals, primary health practitioners and researchers are included with those of policy makers in addressing such structural changes to strengthen service delivery. Future research, policy, and practice must prioritise active engagement and ongoing collaboration among all stakeholders to drive meaningful improvements in post-diagnostic support.

## Note

1. For the purposes of this paper, post-diagnostic support is defined as encompassing both pharmacological and non-pharmacological interventions, as well as support programs provided to individuals with dementia and their carers after diagnosis (Pavković et al., 2024). However, since participants in this study often used “care” to refer to medical care and pharmacological therapy, and “support” to refer to advice, information, or support programs, these terms are also used.

## Appendix

### The Questions Asked in the Semi-structured Interviews Were

• Please share any details about your health concerns, particularly regarding memory problems, and your experiences with visiting a doctor for these issues.
• How satisfied are you with the communication within the memory clinics you’ve attended?
• Please share any advice or information provided by doctors or staff regarding the treatment and management of your memory problems.
• Please, tell us about any written materials you were given, and what information you received.
• Please share any services you have used provided by memory clinics or available in your local area for support or therapy, such as exercise programs or other interventions.
• What aspects of your life do you find most significant or challenging in dealing with your or your partner’s memory problems?
• In what areas do you feel there could be room for improvement?
• Please share any suggestions or ideas on how your life could be enhanced or improved given your experiences with memory issues.
